# Does reduction in mycophenolic acid dose compromise efficacy regardless of tacrolimus exposure level? An analysis of prospective data from the Mycophenolic Renal Transplant (MORE) Registry

**DOI:** 10.1111/j.1399-0012.2012.01694.x

**Published:** 2012-08-02

**Authors:** Anthony Langone, Cataldo Doria, Stuart Greenstein, Mohanram Narayanan, Kimi Ueda, Bashir Sankari, Oleh Pankewycz, Fuad Shihab, Laurence Chan

**Affiliations:** a Vanderbilt University Medical CenterNashville, TN, USA; b Thomas Jefferson University HospitalPhiladelphia, PA, USA; c Montefiore Medical CenterNew York, NY, USA; d Scott and White Memorial HospitalTemple, TX, USA; e California Pacific Medical CenterSan Francisco, CA, USA; f Charleston Area Medical CenterCharleston, WV, USA; g State University of New York at BuffaloBuffalo, NY, USA; h University of Utah School of MedicineSalt Lake City, UT, USA; i University of Colorado Medical CenterDenver, CO, USA

**Keywords:** cellcept, EC-MPS, kidney transplantation, mycophenolate mofetil, mycophenolate, myfortic, outcomes, tacrolimus

## Abstract

Prospective data are lacking concerning the effect of reduced mycophenolic acid (MPA) dosing on efficacy and the influence of concomitant tacrolimus exposure. The Mycophenolic Renal Transplant (MORE) Registry is a prospective, observational study of de novo kidney transplant patients receiving MPA therapy under routine management. The effect of MPA dose reduction, interruption, or discontinuation (dose changes) was assessed in 870 tacrolimus-treated patients: 375 (43.1%) reduced tacrolimus (≤7 ng/mL at baseline) and 495 (56.9%) standard tacrolimus (>7 ng/mL); enteric-coated mycophenolate sodium 589 (67.7%) and mycophenolate mofetil 281 (32.3%). During baseline to month 1, months 1–3, months 3–6, and months 6–12, 9.3% (78/838), 16.6% (132/794), 20.7% (145/701), and 13.1% (70/535) patients, respectively, required MPA dose changes. These patients experienced an increased risk of biopsy-proven acute rejection at one yr with tacrolimus exposure either included in the model (hazard ratio [HR] 2.60, 95% CI 1.28–5.29, p = 0.008) or excluded (HR 2.58, 95% CI 1.28–5.23, p = 0.008). MPA dose changes were significantly associated with one yr graft failure when tacrolimus exposure was included (HR 2.23; 95% CI 1.01–4.89, p = 0.047) but not when tacrolimus exposure was excluded (HR 2.16; 95% CI 0.99–4.79; p = 0.054). These results suggest that reducing or discontinuing MPA can adversely affect graft outcomes regardless of tacrolimus trough levels.

Mycophenolic acid (MPA) is a routine component of immunosuppression regimens following kidney transplantation (1). However, dose-dependent toxicity frequently necessitates reduction or interruption of MPA dosing (2–8). Retrospective data indicate that leukopenia is the most frequent trigger for MPA dose reduction in kidney transplantation, followed by gastrointestinal adverse events and infection (3). Such changes appear to have a marked effect on transplant outcomes. Mycophenolate mofetil (MMF) reduction or discontinuation is associated with a significant increase in acute rejection (3, 9) and graft loss (10) in kidney transplant patients. Large-scale retrospective analyses of registry data from the United States (5, 8) and Europe (11) have also demonstrated a significantly greater risk of kidney allograft loss following cessation of MMF (5, 8, 11) or a dose reduction of 50% or more (5, 8).

Current data, however, are subject to limitations. First, prospective data of reduced-dose MPA are limited to small single-center trials (12, 13). Second, little is known about the relationship between changes to MPA dosing and concomitant exposure to calcineurin inhibitors (CNIs). It has not been determined whether reduction or elimination of MPA treatment confers the same risk in patients receiving reduced-exposure or standard-exposure CNI therapy. Moreover, the available data are largely derived from studies in which either cyclosporine or mixed CNI therapy (cyclosporine or tacrolimus) was administered, whereas many centers now routinely use tacrolimus-based immunosuppression. Third, evidence is sparse concerning the comparative effect of the MMF and enteric-coated mycophenolate sodium (EC-MPS) formulations. While efficacy and safety of the two formulations were similar in pivotal trials (14, 15), subsequent trials using patient-reported outcomes have suggested that conversion from MMF to EC-MPS may improve the gastrointestinal symptom burden (16–18) with the potential to minimize dose reductions or discontinuations (19) and permit maintenance of higher MPA dosing (20–23).

The Mycophenolic Renal Transplant (MORE) Registry prospectively collects data on kidney transplant patients receiving MPA and tacrolimus at the time of transplantation, followed to five yr post-transplant. The purpose of the current analysis was to test the hypothesis that reduction, interruption, or discontinuation of MPA compromises immunosuppressive efficacy regardless of tacrolimus exposure. Specifically, data were analyzed to determine whether such changes to MPA dosing are associated with increased risks of biopsy-proven acute rejection (BPAR) and early graft failure and whether this association is influenced by the extent of exposure to tacrolimus.

## Material and methods

### Study design

The MORE Registry is a five-yr, international, prospective, observational study of de novo renal transplant patients receiving MPA therapy (either EC-MPS or MMF). Eligible sites were selected to meet geographic and size diversity. The study is performed under routine clinical conditions according to local practice. Recruitment started in June 2007 and closed in May 2010. Data collection is ongoing. The MORE Registry is conducted according to the Declaration of Helsinki, and informed consent is obtained for all enrolled patients.

### Study population

Adult (≥18 yr) recipients of a deceased or living donor kidney transplant administered MPA (EC-MPS or MMF) prior to hospital discharge were eligible for enrollment. Patients were excluded if they had received or planned to receive a bone marrow or other solid organ transplant, if they were enrolled or were planned to enroll into an investigational clinical trial involving an immunosuppressive agent that is either blinded or unapproved by the Food and Drug Administration, or if they are unlikely to complete five yr of follow-up. To minimize the possibility of center-imposed bias, investigators at each site agreed to seek participation of all eligible de novo renal transplant recipients seen at the study site within two wk of transplantation. However, enrollment to the registry was capped at a ratio of EC-MPS /MMF recipients of approximately 2:1. The current analysis was restricted to patients who were receiving tacrolimus maintenance immunosuppression at baseline, as this group represented 95% of patients in the MORE Registry.

### MPA therapy

The type of MPA utilized (EC-MPS or MMF) and any dose adjustments were determined by center-specific protocols.

### Data collection and evaluation

Data are recorded at baseline (defined as within two wk of transplantation) and at specified intervals based on routine post-transplantation clinic visits, that is, months 1, 3, 6, and 12 and annually thereafter to five yr. Only data already available to the site clinical team are collected. Information is entered by designated investigator staff to a web-based electronic data capture system with real-time data validation checks to ensure data quality. Data undergo an automated data quality review followed by data management review and both electronic and on-site monitoring.

At each post-baseline study visit, data collection includes occurrence of BPAR, graft and patient survival status, type of immunosuppression, laboratory results, and adverse events. Any reduction, interruption, or discontinuation of MPA dose since the previous visit was recorded and included under the general term “MPA dose change.” The reason for MPA dose change was recorded. EC-MPS dose was converted to the MMF equivalent by multiplying the EC-MPS dose by 1.3889 (24). Tacrolimus trough level was recorded at each clinic visit, using immunoassay-based methods in >89% of cases at each time point. Patients were categorized as receiving reduced (≤7 ng/mL) or standard (>7 ng/mL) tacrolimus exposure (25). Serum creatinine at baseline and at months 1, 3, 6, and 12 was used to calculate estimated glomerular filtration rate (eGFR) using the abbreviated Modification of Diet in Renal Disease four-variable formula (26).

### Statistical methods

Data recorded to June 2010 are presented for the first year post-transplant. All analyses were performed using data pooled across centers and are exploratory. Between-group comparisons were performed based on analysis of variance (ANOVA) for continuous variables and Pearson's chi-square test for categorical variables. Cox proportional hazards modeling was used to analyze adjusted risk of first episode of BPAR and graft failure, with MPA dose change included in the models as a time-varying covariate. To evaluate the impact of tacrolimus exposure on the effect of MPA dose change, two multivariable regression models were fit to the data: one included tacrolimus exposure as a time-dependent covariate, and one did not include tacrolimus exposure. A third model, in which only tacrolimus exposure was included, was also fit to the data. All regression models included recipient age and donor type (living vs. deceased donor). Regression models for BPAR and eGFR also included baseline MPA dose (per mg), recipient race and gender, primary indication for renal transplantation, baseline serum creatinine (per mg/dL), and donor age. Because of low event rates, these factors were not included in the multivariable Cox proportional hazards model for graft failure. Missing data were not imputed in any of the analyses. Analyses were performed using SAS statistical software (SAS Institute, Cary, NC, USA). p < 0.05 was considered statistically significant.

## Results

In total, 870 patients from 40 centers received tacrolimus at the time of transplant, provided baseline tacrolimus trough concentration data, and formed the analysis population. The mean (SD) duration of follow-up was 429 (280) d (median 370, range 10–1107 d). The majority of patients were men (63.8%, 555/870), with a mean age of approximately 52 yr, and fewer than 9.0% of patients (78/870) had undergone a previous kidney transplant.

### Tacrolimus immunosuppression

**Fig. 1:**
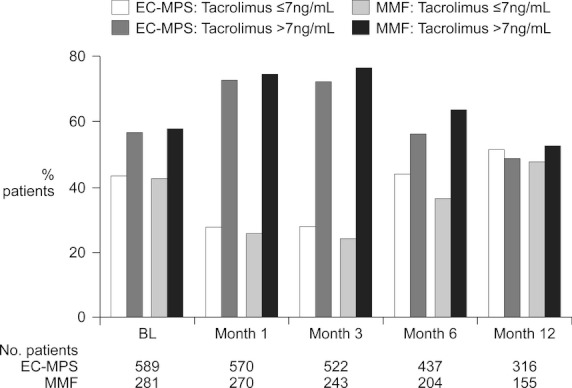
Proportion of patients receiving reduced tacrolimus (trough concentration ≤7 ng/mL) and standard tacrolimus (trough concentration >7 ng/mL) at baseline (i.e., within two wk of transplantation) and months 1, 3, 6, and 12.

At baseline, 375 patients (43.1%) were receiving reduced tacrolimus exposure (≤7 ng/mL), and 495 (56.9%) were receiving standard exposure (>7 ng/mL). There was a slightly older recipient age, more white donors, a lower rate of pre-transplant dialysis, and more living donors with fewer donations after cardiac death in the standard-exposure group (Table 1). As would be expected, the proportion of patients with tacrolimus trough concentration >7 ng/mL peaked at months 1 and 3 post-transplant (Fig. 1).

**Table 1:** Recipient and donor characteristics by baseline tacrolimus exposure and type of MPA treatment

	Baseline tacrolimus exposure^a^			Baseline MPA		
		
	Reduced tacrolimus N = 375	Standard tacrolimus N = 495	p Value^b^	EC-MPS N = 589	MMF N = 281	p Value^b^
Follow-up (d)						0.034
Mean (SD)	410 (266)	443 (289)		415 (271)	459 (296)	
Median	365	386		367	388	
Range	10–1107	11–1094		10–1107	14–1094	
Recipient						
Age (yr), mean (SD)	50.6 (13.5)	52.5 (13.4)	0.039	52.4 (13.4)	50.2 (13.5)	0.025
Male gender, n (%)	246 (65.6)	309 (62.4)		370 (62.8)	185 (65.8)	
Race/ethnicity, n (%)^c^			<0.001^d^			
White	236 (62.9)	355 (71.7)		400 (67.9)	191 (68.0)	
African American	116 (30.9)	92 (18.6)		143 (24.3)	65 (23.1)	
Other	28 (7.5)	50 (10.1)		50 (8.5)	28 (10.0)	
Previous renal transplant, n (%)	34 (9.1)	44 (8.9)		54 (9.2)	24 (8.5)	
Pre-transplant dialysis, n (%)	317 (84.5)	391 (79.0)	0.038	484 (82.2)	224 (79.7)	
Reason for transplantation, n (%)						
Hypertension/nephrosclerosis	89 (23.7)	110 (22.2)		140 (23.8)	59 (21.0)	
Diabetes mellitus	83 (22.1)	119 (24.0)		133 (22.6)	69 (24.6)	
Polycystic disease	46 (12.3)	54 (10.9)		71 (12.1)	29 (10.3)	
Glomerulonephritis/glomerular disease	52 (13.9)	73 (14.8)		93 (15.8)	32 (11.4)	
Other	90 (24.0)	114 (23.0)		122 (20.7)	82 (29.2)	
Unknown	15 (4.0)	25 (5.1)		30 (5.1)	10 (3.6)	
Peak panel-reactive antibody <30%, n/N,%	274/329 (83.3)	368/445 (82.7)		433/523 (82.8)	209/251 (83.3)	
Delayed graft function, n/N (%)	53/360 (14.7)	78/485 (16.1)		85/574 (14.8)	46/271 (17.0)	
Donor						
Age (yr), mean (SD)	42.2 (14.7)	40.7 (14.6)		41.3 (14.8)	41.5 (12.3)	
Age ≥60 yr, n (%)	33 (8.9)	47 (9.6)		56 (9.6)	24 (8.6)	
Male gender, n (%)	197 (52.5)	244 (49.5)		316 (53.7)	125 (44.6)	0.012
Type of donor, n/N (%)			0.017^d^			
Deceased (heart beating)	160/374 (42.8)	223/494 (45.1)		272/587 (46.3)	111/281 (39.5)	
Donation after cardiac death	66/374 (17.6)	51/494 (10.3)		84/587 (14.3)	33/281 (11.7)	
Living related	82/374 (21.9)	117/494 (23.7)		127/587 (21.6)	72/281 (25.6)	
Living unrelated	66/374 (17.6)	103/494 (20.9)		104/587 (17.7)	65/281 (23.1)	
Expanded criteria donor, n/N (%)	49/374 (13.1)	56/492 (11.4)		73/587 (12.4)	32/279 (11.5)	
Cold ischemia time (h), mean (SD)	11.0 (9.9)	10.3 (10.1)		11.0 (9.9)	9.8 (10.3)	

MPA, mycophenolic acid; EC-MPS, enteric-coated mycophenolate sodium; MMF, mycophenolate mofetil; SD, standard deviation.

All differences were non-significant unless stated otherwise. Significant p values (<0.05) are shown.

a Within two wk post-transplant.

b p Value is based on analysis of variance for continuous variables and Pearson's chi-square test for categorical variables.

c Patients could be entered in more than one category.

d p Value refers to a comparison across all categories.

Patients receiving reduced tacrolimus exposure received a significantly lower dose of MPA at baseline, were less likely to receive corticosteroid induction therapy, were less likely to receive rabbit antithymocyte antibody induction, and were more likely to receive alemtuzumab induction, compared with those given a standard tacrolimus regimen (Table 2).

**Table 2:** Immunosuppression at baseline according to tacrolimus exposure and type of MPA treatment at baseline^a^

	Baseline tacrolimus exposure^a^			Baseline MPA treatment^a^		
		
	Reduced tacrolimus N = 375	Standard tacrolimus N = 495	p Value	EC-MPS N = 589	MMF N = 281	p Value
Tacrolimus						
Dose (mg/d)	6.4 (3.9)	6.6 (3.8)	0.479	6.3 (3.9)	6.9 (3.6)	0.018
Trough concentration (ng/mL)	4.4 (1.7)	11.9 (4.1)	<0.001	8.6 (4.8)	8.9 (5.2)	0.303
EC-MPS^b^						
n	256 (68.3)	333 (67.3)		589 (100.0)	–	
Dose (MMF equivalent)^b^ (mg/d)	1806 (471)	1909 (309)	0.002	1864 (391)	–	0.599
<2 g/d, n (%)	65 (25.4)	42 (12.7)		107 (18.2)	–	
2 g/d, n (%)	183 (71.5)	287 (86.5)		470 (79.9)	–	
>2 g/d, n (%)	8 (3.1)	3 (0.9)		11 (1.9)	–	
MMF						
n	119 (31.7)	162 (32.7)		–	281 (100.0)	
Dose (mg/d)	1771 (470)	1906 (382)	0.009	–	1849 (426)	0.599
<2 g/d, n (%)	39 (32.8)	26 (16.1)		–	65 (23.1)	
2 g/d, n (%)	76 (63.9)	130 (80.3)		–	206 (73.3)	
>2 g/d, n%)	4 (3.4)	6 (3.7)		–	10 (3.6)	
Corticosteroids						
Induction, n (%)	315 (84.0)	440 (88.9)	0.0350	508 (86.3)	247 (87.9)	0.501
Maintenance, n (%)	214 (57.1)	286 (57.8)	0.834	349 (59.3)	151 (53.7)	0.124
Dose (mg/d)	33.8 (50.4)	36.7 (51.8)	0.532	35.6 (53.8)	35.1 (44.6)	0.923
Induction therapy, n (%)						
IL-2 receptor antibody	100 (26.7)	119 (24.0)	0.377	144 (24.5)	75 (26.7)	0.476
Antithymocyte antibody (rabbit)	204 (54.4)	329 (66.5)	<0.001	376 (63.8)	157 (55.9)	0.024
Alemtuzumab	59 (15.7)	38 (7.7)	<0.001	56 (9.5)	41 (14.6)	0.026

EC-MPS, enteric-coated mycophenolate sodium; MMF, mycophenolate mofetil; MPA, mycophenolic acid; SD, standard deviation.

Continuous variables are shown as mean (SD).

a Within two wk post-transplantation. Two outliers (tacrolimus dose recorded as 1080 and 360 mg/d were excluded).

b EC-MPS dose was converted to the MMF equivalent by multiplying the EC-MPS dose by 1.3889 (24).

### MtPA immunosuppression

Approximately two-thirds of patients (n = 589, 67.7%) were receiving EC-MPS at baseline; the remaining 281 patients (32.3%) received MMF. Compared to the MMF-treated cohort, patients receiving EC-MPS had a significantly shorter follow-up period (reflecting more recent adoption of the newer formulation), were slightly older, and were more likely to receive a graft from a donor aged 60 yr or older (Table 1). The proportion of patients with a baseline dose <2 g/d (in MMF equivalent doses) was 18.2% (107/589) in the EC-MPS group and 23.1% (65/281) in the MMF group (n.s.); mean baseline dose was similar with either formulation. Concomitant immunosuppression, including tacrolimus exposure (Fig. 1), was similar in the MMF and EC-MPS groups other than a greater incidence of rabbit antibody induction in the EC-MPS cohort and a greater incidence of alemtuzumab induction in the MMF cohort (Table 2).

The mean dose of EC-MPS (in MMF equivalent doses) and MMF was 1850 ± 381 and 1789 ± 443 mg/d at month 1 (p = 0.044), 1752 ± 469 and 1657 ± 493 mg/d at month 3 (p = 0.011), 1594 ± 518 and 1506 ± 533 mg/d at month 6 (p = 0.051), and 1545 ± 507 and 1448 ± 540 mg/d at month 12 (p = 0.057). Post-baseline, 10 patients switched from EC-MPS to MMF: the reasons were economic (2), acute rejection (2), optimization of therapy (1), administrative (1), and other (4). Thirty-three patients switched from MMF to EC-MPS owing to gastrointestinal adverse events (23), viral adverse event (1), hematologic event (1), or economic reasons (1); the reason for switch was missing for seven patients.

During the periods baseline to month 1, months 1–3, months 3–6, and months 6–12, 9.3% (78/838), 16.6% (132/794), 20.7% (145/701), and 13.1% (70/535) patients, respectively, required one or more MPA dose change. The most frequent modification was MPA dose reduction (69–85% of cases [Table 3]). There were no significant differences in the proportion of EC-MPS- or MMF-treated patients requiring a MPA dose change at any period during the first 12 months post-transplant, or any marked variation in the causes (Table 3). Patients who required a MPA dose change had a lower mean tacrolimus trough concentration at all time points, which was significant at month 1 (p = 0.047) and month 12 (p = 0.014), and received a slightly lower dose of corticosteroids than those in whom the initial MPA dose remained unchanged (Table 4).

**Table 3:** Incidence and causes of MPA dose changes by time post-transplantation according to type of MPA treatment at baseline, n (%)

	EC-MPS				MMF			
		
	Baseline to month 1 N = 569	Months 1–3 N = 537	Months 3–6 N = 474	Months 6–12 N = 358	Baseline to Month 1 N = 269	Months 1–3 N = 257	Months 3–6 N = 227	Months 6–12 N = 177
MPA dose change	58 (10.2)	85 (15.8)	103 (21.7)	47 (13.1)	20 (7.4)	47 (18.3)	42 (18.5)	23 (13.0)
MPA reduced	49 (84.5)	66 (77.7)	77 (74.8)	34 (72.3)	17 (85.0)	38 (80.9)	29 (69.1)	18 (78.3)
MPA interrupted	3 (5.2)	6 (7.1)	8 (7.8)	4 (8.5)	2 (10.0)	0	5 (11.9)	0
MPA discontinued	6 (10.3)	13 (15.3)	18 (17.5)	9 (19.2)	1 (5.0)	9 (19.2)	8 (19.1)	5 (21.7)
p Value for MPA dose change EC-MPS vs. MMF					0.200	0.384	0.324	0.966
Reason(s)^a^								
Hematologic^b^	20 (3.5)	44 (8.2)	54 (11.4)	14 (3.9)	8 (3.0)	29 (11.3)	27 (11.9)	9 (5.1)
Gastrointestinal adverse event^b^	35 (6.2)	30 (5.6)	24 (5.1)	15 (4.2)	11 (4.1)	17 (6.6)	5 (2.2)	3 (1.7)
Viral adverse event^b^	4 (1.7)	15 (2.8)	28 (5.9)	16 (4.5)	1 (0.4)	4 (1.6)	13 (5.7)	11 (6.2)
CMV infection^b^	1 (0.2)	4 (0.7)	7 (1.5)	7 (2.0)	0	1 (0.4)	3 (1.3)	4 (2.3)
BKV infection^b^	3 (0.5)	12 (2.2)	22 (4.6)	9 (2.5)	1 (0.4)	3 (1.2)	11 (4.8)	8 (4.5)
Acute rejection	1 (0.2)	0	2 (0.4)	2 (0.6)	1 (0.4)	0	2 (0.9)	2 (1.1)
Chronic allograft nephropathy	0	0	0	0	0	0	0	0
Bone-related adverse event	0	1 (0.2)	0	1 (0.3)	0	1 (0.4)	0	0
Nephrotoxicity	0	0	0	0	0	0	0	0
Diabetes mellitus	0	0	0	1 (0.3)	0	0	0	0
Compliance	0	0	1 (0.2)	0	0	0	0	0

EC-MPS, enteric-coated mycophenolate sodium; MMF, mycophenolate mofetil; MPA, mycophenolic acid; BKV, BK polyoma virus; CMV, cytomegalovirus.

Month 1 = reporting period between baseline and month 1; month 3 = reporting period between month 1 and month 3; month 6 = reporting period between month 3 and month 6; month 12 = reporting period between month 6 and month 12.

a More than one reason could be indicated as the reason for a MPA dose change.

b All differences between EC-MPS and MMF were non-significant at all time points. p Values were not calculated for less-frequent reasons.

**Table 4:** Tacrolimus dose and levels and corticosteroid dose by MPA dose changes at 1, 3, 6, and 12 months post-transplant

		Tacrolimus^b^		
			
	MPA dose since last visit^a^	Dose (mg/d)	Trough concentration (ng/mL)	Corticosteroids Dose (mg/d)
Month 1	No dose change	7.0 (4.7) n = 751	9.7 (4.7) n = 746	15.6 (24.3) n = 475
	Dose change	7.1 (4.6) n = 77	8.6 (3.2) n = 77	15.2 (6.7) n = 29
	p Value^c^	0.861	0.047	0.928
Month 3	No dose change	6.2 (4.1) n = 634	9.3 (3.9) n = 624	10.1 (25.2) n = 393
	Dose change	6.7 (4.2) n = 129	9.0 (3.7) n = 127	8.7 (8.5) n = 56
	p Value^c^	0.225	0.310	0.684
Month 6	No dose change	6.0 (3.6) n = 511	8.1 (3.2) n = 498	6.6 (4.6) n = 325
	Dose change	5.6 (3.5) n = 138	7.7 (3.2) n = 133	6.6 (3.7) n = 87
	p Value^c^	0.174	0.213	0.956
Month 12	No dose change	5.6 (3.6) n = 413	7.7 (3.2) n = 402	6.5 (6.9) n = 263
	Dose change	5.3 (2.9) n = 63	6.7 (2.1) n = 61	6.2 (7.7) n = 52
	p Value^c^	0.570	0.014	0.751

MPA, mycophenolic acid.

Doses are shown as mean (SD). MPA dose change included dose reduction, interruption, or discontinuation.

a For patients in whom tacrolimus exposure was known at each time point.

b Outliers excluded at months 1, 6 and 12.

c For MPA dose vs. no MPA dose change.

### Efficacy

The unadjusted incidence of BPAR from baseline to month 1, months 1–3, months 3–6, and months 6–12 was 4.7% (39/823), 1.3% (10/751), 1.3% (8/631), and 2.4% (11/463), respectively, among patients for whom tacrolimus exposure was known at each time point (Table 5). Twelve-month risk of BPAR was 9.3% (95% CI 7.1–11.4%) based on Kaplan–Meier estimates. A further three episodes of BPAR were reported, which were not confirmed by biopsy. Multivariate analysis showed a significantly higher risk of BPAR in patients with a MPA dose change that was unaffected by the inclusion of tacrolimus exposure as a covariate (Table 6) that is, patients with a MPA dose change were at a similarly increased risk of BPAR regardless of concomitant tacrolimus trough level. Consistent with this, the unadjusted incidence of BPAR in patients with reduced or standard tacrolimus exposure was similar for patients who did or did not require a MPA dose change (Table 5), and there was no significant association between tacrolimus exposure and risk of BPAR after adjustment for confounding variables (hazard ratio [HR] 0.83, 95% CI 0.45–1.53, p = 0.545 [Table 6]).

**Table 5:** Unadjusted incidence of BPAR according to MPA dose changes and baseline tacrolimus exposure, n/N (%)^a^ MPA dose change included dose reduction, interruption, or discontinuation

	No MPA dose change			MPA dose change		
		
	Reduced tacrolimus	Standard tacrolimus	All	Reduced tacrolimus	Standard tacrolimus	All
Baseline to Month 1	22/554 (4.0)	11/192 (5.7)	33/746 (4.4)	3/46 (6.5)	3/31 (9.7)	6/77 (7.8)
Months 1–3	3/460 (0.7)	3/164 (1.8)	6/624 (1.0)	3/90 (3.3)	1/37 (2.7)	4/127 (3.2)
Months 1–6	1/292 (0.3)	5/206 (2.4)	6/498 (1.2)	1/77 (1.3)	1/56 (1.8)	2/133 (1.5)
Months 6–12	3/207 (1.5)	5/195 (2.6)	8/402 (2.0)	0/24 (0)	3/37 (8.1)	3/61 (4.9)

MPA, mycophenolic acid; BPAR, biopsy-proven acute rejection.

a For patients in whom tacrolimus exposure was known at each time point.

**Table 6:** Risk of BPAR at one yr and graft loss at one yr after adjustment for confounding variables (Cox regression analysis)

	HR	95% CI	p Value
BPAR at one yr			
MPA dose change			
Tacrolimus exposure included as a covariable	2.60	1.28–5.29	0.008
Tacrolimus exposure not included as a covariable	2.58	1.28–5.23	0.008
Reduced tacrolimus exposure	0.83	0.45–1.53	0.545
Graft loss at one yr			
MPA dose change			
Tacrolimus exposure included as a covariable	2.23	1.01–4.89	0.047
Tacrolimus exposure not included as a covariable	2.16	0.99–4.79	0.054
Reduced tacrolimus exposure	1.17	0.56–2.44	0.684

HR, hazard ratio; CI, confidence interval; MPA, mycophenolic acid; BPAR, biopsy-proven acute rejection.

MPA dose change included dose reduction, interruption, or discontinuation.

Twelve-month graft survival was 95.6% (95% CI 94.0–97.2%) based on Kaplan–Meier estimates. Cox regression modeling showed MPA dose change to be associated with more than a twofold increase in risk of graft failure at one yr, an association that was significant when tacrolimus exposure was included as a covariable in the model (Table 6). There was no evidence of a statistically significant association between tacrolimus exposure and risk of graft failure at one yr (HR for reduced exposure vs. standard exposure 1.17; 95% CI 0.56–2.44; p = 0.684).

### Renal function

eGFR was significantly higher in patients with no change in MPA dose at month 1: mean 54.8 (19.0) mL/min/1.73 m^2^ vs. 45.1 (17.6) mL/min/1.73 m^2^ in patients requiring a MPA dose change (p < 0.001), but similar between groups thereafter. Baseline eGFR was significantly higher in the standard-tacrolimus group (43.7 [25.5] mL/min/1.73 m^2^) than in the reduced-tacrolimus group (37.2 [43.7] mL/min/1.73 m^2^, p = 0.0004). However, eGFR was subsequently similar between groups: mean (SD) values at 1, 3, 6, and 12 months post-transplantation were 53.2 (18.7), 56.8 (18.4), 59.0 (20.2), and 59.5 (20.3) mL/min/1.73 m^2^ in the reduced-exposure group, respectively, vs. 52.2 (19.6), 56.2 (18.1), 57.5 (18.7), and 56.4 (19.6) mL/min/1.73 m^2^ for the standard-exposure group (p = n.s. at each time point).

## Discussion

In this first prospective analysis of the impact of MPA dosing on graft outcomes following kidney transplantation, reduction, interruption, or discontinuation of MPA treatment was associated with more than a twofold increase in the risk of both BPAR and graft failure. Notably, the risk that patients would require a MPA dose change was unaffected by concomitant tacrolimus exposure.

To our knowledge, the current analysis is the first to show that the increased risk of BPAR in patients with MPA dose reduction, interruption, or discontinuation is not influenced by whether the patient is receiving standard or reduced tacrolimus exposure. Patients who did not continue to receive their initial MPA dose experienced more than a 2.5-fold increase in adjusted risk of BPAR vs. those in whom MPA dose remained unchanged, a difference that was virtually unaffected by the inclusion of tacrolimus exposure in the model. In an early dose-finding study of MMF, in which kidney transplant patients were given doses of between 1000 mg/d and 3.5 g/d, there was a clear correlation between MMF dose and incidence of rejection (27), and previous retrospective studies have confirmed that MPA dose reductions or discontinuation incur a higher risk of rejection (3, 9), but concomitant CNI concentrations were not described. Evidence from clinical trials (25, 28, 29) indicates that reduced CNI exposure does not influence rejection rates in MPA-treated patients but, conversely, changes to MPA dosing have not been reported. The recent Symphony study observed no difference in the one-yr (25) or three-yr (28) incidence of BPAR in kidney transplant patients receiving reduced or standard cyclosporine exposure in combination with MMF. Similarly, a meta-analysis of 19 randomized studies undertaken in kidney transplant patients receiving MPA found that acute rejection rates were not affected by CNI exposure reduction (29), consistent with data from retrospective series (30, 31).

The risk of graft failure was also significantly increased by more than twofold in patients who required a MPA dose change, regardless of tacrolimus level. Adequate MPA dosing appears essential for optimal graft outcomes. Retrospective registry analyses have previously shown significantly higher rates of graft loss following cessation of MMF (an increase of between 1.5- and 3.2-fold [5, 8, 11]) or a dose reduction of 50% or more (1.3–2.4-fold) (5, 8, 11), consistent with our results. Here, the absolute rate of BPAR was relatively low (7.8% in patients for whom the MPA dose was changed by month 1), and although data on the severity of BPAR were not consistently captured and cannot be analyzed reliably, mild episodes are likely to have accounted for at least some of the reported rejection events and may have had a relatively minor effect on subsequent graft survival (32, 33). Nevertheless, the differences in BPAR between groups appear to account for the variation in graft survival rates.

Hematologic adverse events were the most frequent cause of MPA dose change during months 1–6, declining thereafter. MPA dose changes in response to BK polyoma virus infection (34) peaked during months 3–6. Gastrointestinal events as a cause of MPA dose changes became less frequent after month 6, possibly due to a lessening of tacrolimus-related gastrointestinal effects (35) as tacrolimus exposure decreased over time. The pattern of MPA dose changes must, however, be interpreted with caution as patients who were most susceptible to complications withdrew from the study throughout follow-up, leaving patients who were progressively less likely to require a MPA dose change remaining in the analysis. No consistent differences between EC-MPS and MMF were observed in terms of either the rate or causes of MPA dose changes, including gastrointestinal events. The pivotal comparative trial of EC-MPS vs. MMF in de novo kidney transplant recipients showed no difference in the frequency of dose changes owing to gastrointestinal intolerance (14), but more sensitive patient-reported outcomes were not used (16–18). Here, the dose of MPA was maintained at a significantly higher level in the EC-MPS group vs. the MMF group during the first three months post-transplant, with a trend to significance thereafter. This is consistent with recent findings from a randomized trial of kidney transplants with gastrointestinal symptoms in which significantly more patients tolerated a dose increase with EC-MPS than with MMF (20). In the current analysis, 23 patients converted from MMF to EC-MPS for gastrointestinal symptoms, but there were no switches from EC-MPS to MMF for this cause.

The strengths of this study include the prospective data collection, the correct use of a time-dependent variable for MPA dose changes and tacrolimus exposure, and a relatively large patient population representative of routine clinical practice. Drug dosage changes and rejection episodes were captured in detail during routine monitoring, although timing of data collection was not necessarily consistent across centers. However, as an observational study, the analysis inevitably has limitations. First, a causal relationship between MPA dosing and efficacy outcomes cannot be established. An interventional trial in which the MPA dose is administered below the recommended level without a medical indication would be considered unethical. Second, transplant centers were free to select immunosuppression according to local protocols, and the potential for bias in patient selection cannot be ruled out. Preferential use of EC-MPS or MMF in particular patients seems unlikely as centers typically have only one MPA treatment on their inpatient hospital formulary. Perhaps more pertinently, indications for MPA dose adjustment and tacrolimus exposure were not protocol-mandated, and reasons for dosing choices are not known. The observation that tacrolimus exposure was lower in patients who required a MPA dose change might reflect an attempt to reduce all drugs known to exert gastrointestinal effects in symptomatic patients, lowering the exposure of all immunosuppressant agents in patients with BK infection, or a more aggressive immunosuppression minimization strategy at certain transplant centers. Similarly, steroid dosing or withdrawal was not protocol-specified. Third, MPA concentrations were not measured, so the effect of MPA exposure on efficacy cannot be assessed, although data regarding the benefit of measuring MPA levels are inconclusive (36–39).

Despite these limitations, the findings of this prospective analysis of patients managed under routine clinical conditions suggest that caution should be exercised when the MPA dose is reduced or discontinued in patients receiving tacrolimus, regardless of the extent of tacrolimus exposure.
